# A phase I, open-label trial on the safety and immunogenicity of the adjuvanted tuberculosis subunit vaccine H1/IC31® in people living in a TB-endemic area

**DOI:** 10.1186/s13063-017-2354-0

**Published:** 2018-01-10

**Authors:** Jemal Hussein, Martha Zewdie, Lawrence Yamuah, Ahmed Bedru, Markos Abebe, Alemnew F. Dagnew, Menberework Chanyalew, Asfawesen G. Yohannes, Jemal Ahmed, Howard Engers, T. Mark Doherty, Peter Bang, Ingrid Kromann, Søren T. Hoff, Abraham Aseffa

**Affiliations:** 1 Armauer Hansen Research Institute (AHRI), Jimma Road, PO Box 1005, Addis Ababa, Ethiopia; 2 Statens Serum Institut (SSI), Artillerivej 5, 2300 Copenhagen, Denmark; 3 KNCV Tuberculosis foundation, Challenge TB project, Addis Ababa, Ethiopia; 4 GlaxoSmithKline Vaccines, Rockville, MD USA; 5 GlaxoSmithKline Vaccines, Wavre, Belgium; 6 Present address: Novo Nordisk, Copenhagen, Denmark

**Keywords:** Tuberculosis, Vaccines, Immunogenicity

## Abstract

**Background:**

H1/IC31® is a tuberculosis (TB) subunit vaccine candidate consisting of the fusion protein of Ag85B and ESAT-6 (H1) formulated with the IC31® adjuvant. Previous trials have reported on the H1/IC31® vaccine in *M. tuberculosis* (Mtb)-naïve, BCG-vaccinated and previously Mtb-infected individuals. In this trial, conducted between December 2008 and April 2010, the safety and immunogenicity of H1/IC31® was assessed in participants living in Ethiopia – a highly TB-endemic area.

**Methods:**

Healthy male participants aged 18–25 years were recruited into four groups. Participants in group 1 (*N* = 12) and group 2 (*N* = 12) were Tuberculin Skin Test (TST) negative and QuantiFERON-TB Gold in-tube test (QFT) negative (Mtb-naïve groups), participants in group 3 (*N* = 3) were TST positive and QFT negative (BCG group), and participants in group 4 (*N* = 12) were both TST and QFT positive (Mtb-infected group). H1 vaccine alone (group 1) or H1 formulated with the adjuvant IC31® (groups 2, 3 and 4) was administered intramuscularly on day 0 and day 56. Safety and immunogenicity parameters were evaluated for up to 32 weeks after day 0.

**Results:**

The H1/IC31®vaccine was safe and generally well tolerated. There was little difference among the four groups, with a tendency towards a higher incidence of adverse events in Mtb-infected compared to Mtb-naïve participants. Two serious adverse events were reported in the Mtb-infected group where a relationship to the vaccine could not be excluded. In both cases the participants recovered without sequelae within 72 h. Immunogenicity assays, evaluated in the 29 participants who received both vaccinations, showed a stronger response to TB antigens in the Mtb-naïve group vaccinated with the adjuvant.

**Conclusion:**

The trial confirmed the need for an adjuvant for the vaccine to be immunogenic and highlighted the importance of early phase testing of a novel TB vaccine candidate in TB-endemic areas.

**Trial registration:**

ClinicalTrials.gov, ID: NCT01049282. Retrospectively registered on 14 January 2010.

**Electronic supplementary material:**

The online version of this article (10.1186/s13063-017-2354-0) contains supplementary material, which is available to authorized users.

## Background

Tuberculosis (TB) remains a global crisis causing active disease in 8.6 million people annually of which 1.3 million die [1]. An estimated two billion people are latently infected with *M. tuberculosis* (Mtb) making up a huge reservoir for new TB cases and continued transmission.

Vaccines are an important tool for control of infectious diseases, especially in resource-poor countries, but the Bacillus Calmette-Guérin (BCG) vaccine is the only vaccine currently available against TB. Neonatal vaccination with BCG is effective against pulmonary as well as disseminated TB disease in infants and children [2–4]. BCG vaccination also confers protection against pulmonary TB in mycobacteria-naïve adults when given as an adult vaccine [3]. In TB-endemic areas the BCG vaccine has shown varying efficacy apparently because of waning efficacy over time, and lack of effect in already-infected or sensitized individuals [2–4]. A novel vaccine against TB, which is safe and effective in both Mtb-naïve individuals and Mtb-infected individuals, is needed for prevention of infection, disease progression and overall reduction of disease transmission [5, 6].

Many novel TB vaccines are under investigation in clinical trials [7]. The H1/IC31® vaccine developed by Statens Serum Institut (SSI) is a fusion protein of the two Mtb antigens Ag85B and 6-kDa early secretory antigenic target (ESAT-6) (H1) formulated with the adjuvant IC31® developed by Valneva SE (formerly Intercell AG). Both Ag85B and ESAT-6 are highly immunogenic Mtb antigens and are thought to be important for the survival of the bacteria once phagocytosed by macrophages during initial infection [8–10]. Ag85B is expressed by BCG (albeit at low levels) whereas ESAT-6, belonging to the family of Mtb proteins within the RD1 region, is not [11]. ESAT-6 is thought to have a unique potential in a vaccine targeting already Mtb-infected individuals [12]. The adjuvant system IC31® contains two components; the cationic polyaminoacid KLK, and the oligodeoxynucleotide ODN1a combined at a ratio of 25 KLK to 1 ODN1a [13].

The H1/IC31® vaccine is intended to be used in an adolescent population and designed to be efficacious in BCG-vaccinated, Mtb-naïve and in Mtb-infected individuals alike. For this purpose, it is vital to address and investigate the safety of the vaccine when given to people who already have an established Mtb infection.

Prior to this trial, H1/IC31® vaccine trials have been reported from three clinical trials. Two clinical phase I trials conducted in the Netherlands reported the vaccine to be safe and immunogenic in Mtb-naïve, BCG-vaccinated individuals and individuals with previously treated Mtb infections [14, 15]. The vaccine was shown to retain immunogenicity for up to 2.5 years after two vaccinations [14, 15]. However, participants included in these trials were living in a TB low-endemic area, and as a logical continuation, the current trial was designed to address primarily the safety and secondly, the immunogenicity of the H1/IC31® vaccine in Mtb-naïve, BCG-vaccinated and Mtb-infected individual living in Ethiopia – a highly TB-endemic area. Accordingly, this study was conducted in Addis Ababa, Ethiopia between December 2008 and April 2010. Subsequent to this trial, the H1/IC31® vaccine was found to be safe and immunogenic in HIV-infected individuals living in Tanzania and South Africa, a study conducted between December 2011 to September 2012 [16], and in a large phase II trial including 240 adolescents from the Cape Town area in South Africa, conducted between September 2012 and December 2013 [17].

We here report the results of a phase I, open-label clinical trial investigating the safety and immunogenicity of H1/IC31® administered in different antigen/adjuvant formulations in Mtb-naïve and Mtb-infected individuals living in Ethiopia.

## Methods

### Ethical considerations

The trial application was reviewed and approved by the Development-Country Committee of the  Danish National Committee on Biomedical Research Ethics; the Institutional Review Board at the investigation site, the Armauer Hansen Research Institute (AHRI) and the All Africa Leprosy Rehabilitation and Training Centre (ALERT) Ethical Review Committee; and the National Research Ethics Review Committee of Ethiopia. It was also reviewed and approved by the Food, Medicine and Health Care Administration and Control Authority of Ethiopia (FMHACA); formerly known as the Ethiopian Drug Administration and Control Authority (DACA). Written informed consent was obtained from all participants. The trial was retrospectively registered at ClinicalTrials.gov (NCT01049282).

### Trial population

This study was conducted at the Armauer Hansen Research Institute, Addis Ababa, Ethiopia between December 2008 and April 2010. All participants were male students between 18 and 55 years of age and healthy, based on medical examination/history, and had signed informed consent and granted authorized persons access to their medical records. Participants were screened and enrolled into four groups. Group-1 and group-2 (Mtb-naive) participants were Mtb-uninfected and BCG-naïve based on a negative Tuberculin Skin Test (TST) and a negative QuantiFERON-TB Gold in-tube test (QFT). Group 3 were Mtb-uninfected but BCG-sensitized based on a positive TST (≥10 mm), with evidence of BCG vaccination more than 2 years prior determined by the presence of a scar or vaccination card, and with a negative QFT test. Finally, group-4 participants were apparently healthy with no signs and/or symptoms of TB but Mtb-infected based on both TST and QFT positivity. A chest X-ray was taken during screening and volunteers with findings consistent with active TB were excluded from enrollment. Additionally, vaccination with any vaccine 3 months before the first vaccination date; use of immune modulating drugs (steroids, immunosuppressive drugs or immunoglobulins); hepatitis B virus (HBV), hepatitis C virus (HCV) or HIV sero-positivity; participation in another clinical trial; known hypersensitivity to any of the vaccine components; and laboratory parameters outside of normal range considered clinically relevant were used as exclusion criteria during enrollment.

### Investigational product

The H1/IC31® TB vaccine is a product of Statens Serum Institut (SSI), Denmark. The vaccine preparation contained either the recombinant H1 fusion protein (Ag85B and ESAT-6) at 50 μg per dose alone or mixed with the adjuvant IC31® (Valneva, Austria) composed of 500 nmol KLK and 20 nmol ODN1a per dose as previously described [14].

### Study design

The study was a single-center, open-label, non-randomized, phase I trial in adult male participants investigating the safety and immunogenicity of the H1/IC31® vaccine in Mtb-naïve and Mtb-infected volunteers living in Ethiopia – a TB-endemic area. Women were not enrolled in this study because of ethical issues in recruiting women of child-bearing potential (WOCBP) where it would be challenging to discuss the use of contraceptives in the study population. The vaccine was administered twice intramuscularly at days 0 and 56. Study participants in group 1 received 50 μg of H1 alone, while groups 2, 3 and 4, received 50 μg H1 mixed with adjuvant IC31®. The trial was a non-randomized trial where Mtb-naïve volunteers were assigned sequentially to group 1 and then to group 2 on a first-come-first-served basis. Primary endpoints were local and systemic adverse events (AEs) and laboratory safety parameters of hematology, biochemistry and urinalysis. The accredited International Clinical Laboratories (ICL), Addis Ababa, Ethiopia, performed all laboratory safety tests in the trial. X-rays were taken at AHRI/ALERT. Safety parameters were accessed on the day of both vaccinations at 1, 2, 7 and 42 days after both vaccinations and finally 6 months after the last vaccination. A Data Safety Monitoring Board (DSMB) was appointed to give recommendations for safety assessment during the trial and an independent trial monitor was assigned. The trial was established as part of EDCTP’s capacity-building activities strengthening the clinical trial research infrastructure at AHRI. Handling and storage of the IMP, ethical and authority approval and information and consent of volunteers were done according to the Declaration of Helsinki/Good Clinical Practice (GCP) standards.

### Cell-mediated and humoral immunogenicity assays

Immunogenicity of the vaccine was evaluated by QFT assay before and 224 days after the first vaccination, as well as by IFN-γ Enzyme Linked Immunosorbent Assay (ELISA) and IgG ELISA before vaccination (day 0) and at study days 7, 42, 63, 98 and 224 post first vaccination (Additional file 1). Despite screening a large number of volunteers, only three participants were enrolled and vaccinated in group 3 (TST positive but QFT negative); therefore, their data was excluded from analysis. Endpoints were detection of IFN-γ concentration in supernatants from whole blood stimulated with Mtb antigens ESAT-6, Culture Filtrate Protein-10 (CFP-10) and TB7.7 (QFT assay), IFN-γ concentration in supernatants from peripheral mononuclear cells (PBMCs) stimulated with vaccine components Ag85B or ESAT-6 peptide pools for 7 days and detection of IgG antibodies to the vaccine antigen H1 in plasma. The immunogenicity tests were performed by the Immunology Research Laboratory at AHRI. Plasma for antibody analysis and PBMCs for analysis of antigen-specific T-cell responses from each time point were isolated and frozen until batch analysis by ELISA. IFN-γ ELISA was done using an optimized kit from U-CyTech, Utrecht and IgG ELISA was performed as previously described [15]. The QFT assay (Qiagen, The Netherlands) was done according the manufacturer’s instructions and the data analyzed using the QuantiFERON-TB Gold Analysis Software. Although IFN-γ Enzyme Linked Immunospot Assay (ELISPOT) assay was performed for some of the samples, the data is omitted due to poor recovery of cells in a high proportion of the frozen samples. There were seven visits for immunogenicity assays and 15 of the 29 participants who received both vaccinations had insufficient cells to perform ELISPOT assay for at least two stimuli in two or more visits.

### Statistical analysis

The trial population was planned to consist of 24 Mtb-naïve (12 in each of groups 1 and 2) and 24 TST-positive volunteers (12 BCG-vaccinated volunteers and 12 latent TB volunteers). Although formal sample size calculation was not performed, the sample size was considered sufficient to give the information necessary to address the trial objectives. All participants who received at least the first dose of the vaccine were included in the safety assessment (*N* = 39). All participants who received both vaccinations were included in the immunogenicity assessment (*N* = 29); however, the immunogenicity data from group 3 was excluded from analysis due to the low number of participants. Statistical analysis was done by SSI and AHRI using GraphPad Prism 6 (GraphPad, San Diego, CA, USA). Comparison between groups was done calculating the area under the curve (AUC) for each individual participant and subsequently grouping the individual AUC values before using Kruskal-Wallis for overall effect and, if significant, the Mann-Whitney test for comparison between individual groups. Results presented in this paper are based on source data and Case Report Forms (CRFs). However, the data was also analyzed by an independent company (JGConsult) and the overall findings support the results obtained by SSI/AHRI (Personal communication with JGConsult).

## Results

### Trial participants

**Fig. 1:**
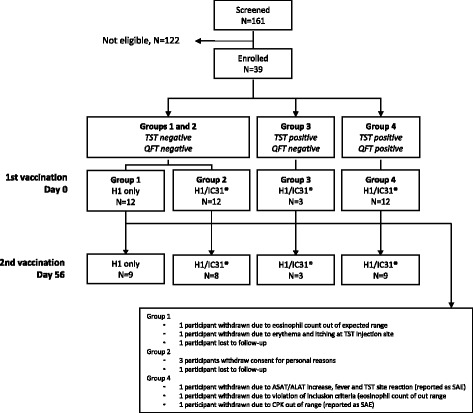
Flow diagram of participant screening, enrollment and vaccination

One hundred and sixty-one persons were screened and 39 participants were included in this study. The reasons for the high number of screening failures seen (*N* = 122) were the large numbers of participants screened in an attempt to find those who met the inclusion criteria for group 3 (TST positive but QFT negative) and the fact that many had eosinophil granulocyte counts outside the expected normal range at screening (Fig. 1). All trial participants were male, aged between 18 and 25 years. The baseline demographic data of the study participants is summarized in Table 1. All 39 participants received the first vaccination, and of these, 29 participants received the second vaccination. Five volunteers withdrew, and five volunteers did not complete all 12 visits in the trial (Fig. 1).

**Table 1:** Baseline demographic and clinical characteristics of study participants

	Total *N* = 39	Group 1 *N* = 12	Group 2 *N* = 12	Group 3 *N* = 3	Group 4 *N* = 12
Age (years)	20	21	20	20	20
Median					
(IQR)	(20–1)	(20–21)	(20–21)	(19–20)	(20–22.5)
BMI	19.4	18.7	19.5	19.1	20.0
Median					
(IQR)	(18.3–20.3)	(17.9–0.3)	(18.4–20.2)	(18.3–21.6)	(18.4–20.7)
QFT test	N/A	Negative	Negative	Negative	Positive
(IU/ml)		0.011	0.038	0.083	11.8

*BMI* Body Mass Index, *IQR* interquartile range, *QFT* QuantiFERON-TB Gold-in-tube test

### Safety

All 39 enrolled participants were included in the descriptive safety analysis (Table 2). A total of 252 adverse events (AEs) were reported; of which 42 were local and 210 systemic AEs. The local AEs occurred in 42%, 25%, 33% and 75% of the participants in groups 1, 2, 3 and 4, respectively, while systemic AEs were noted in 92%, 75%, 67% and 100% of the participants in groups 1, 2, 3 and 4, respectively. Two serious adverse events (SAEs) were reported during the trial. Both were reported in group 4 after first vaccination and considered as possibly vaccine related. Both participants in question were withdrawn from the trial prior to administration of the second vaccination (Fig. 1). The first SAE concerned a participant who had an elevation of liver function tests (AST/ALT) 48 h post first vaccination (AST 214 IU/L from a baseline of 37 IU/L and ALT 397 IU/L from a baseline of 27 IU/L). The participant’s liver function tests improved to AST 108 IU/L and ALT 256 IU/L at follow-up analysis the day after. Concurrent with the elevated ALT/AST, the volunteer complained of fever, had chills, weakness, headache and generalized body weakness. In addition, he had erythema at a previous purified protein derivative (PPD) injection site 12 h post vaccination, which disappeared completely at the day of elevated ALT/AST. The participant had received diclofenac sodium by injection intramuscularly and per os, taken at the day of the event and the day before. The participant recovered without sequelae. The second SAE concerned a participant who had an elevation of CPK to 10,545 IU/L at 24 h post first vaccination and 11,025 IU/L at 48 h from a baseline of 529 IU/L. The participant was an athletic person who used to exercise by weight lifting regularly, but was advised to abstain from this activity for a limited time period. The participant recovered without sequelae and with normalized CPK values within 72 h. Excluding the two participants with out-of-range laboratory blood parameters reported as SAEs, 14 other participants had at least one blood parameter out of range during the trial, the most common being a platelet count below the normal range seen in a total of four participants. Main local AEs were pain, swelling, erythema or itching at the injection sites. The main reported systemic AEs were headache, sediment in the urine, fever and sweating. There was little difference between the four trial groups for most of the reported AEs, although there appeared to be a tendency towards more participants in group 4 experiencing local AEs. Notably, local AEs at the site of prior TST injection were mainly reported in group 4. In summary, with the exception of the two SAEs in the study group with prior TB exposure, most reported AEs were mild, a few were moderate, and in general the AEs resolved within a week.

**Table 2:** Reported local and systemic adverse events (AEs)

	Group 1	Group 2	Group 3	Group 4
TST/QFT at inclusion	Neg/Neg	Neg/Neg	Pos/Neg	Pos/Pos
Vaccination	H1 only	H1/IC31®	H1/IC31®	H1/IC31®
Number of participants	12	12	3	12
	*n*^a^ (%) total AEs	*n* (%) total AEs	*n* (%) total AEs	*n* (%) total AEs
Local AEs				
Any local AE	5 (42) 9	3 (25) 9	1 (33) 4	9 (75) 20
Injection site pain	0 (0) 0	3 (25) 6	1 (33) 1	4 (33) 5
Injection site swelling	1 (8) 1	2 (17) 2	1 (33) 1	1 (8) 1
Injection site erythema	2 (17) 2	0 (0) 0	1 (33) 1	2 (17) 2
Injection site Itching	3 (25) 3	0 (0) 0	1 (33) 1	0 (0) 0
Injection site numbness	0 (0) 0	0 (0) 0	0 (0) 0	2 (17) 2
Injection site stiffness	0 (0) 0	0 (0) 0	0 (0) 0	2 (17) 2
Injection site rash	0 (0) 0	1 (8) 1	0 (0) 0	0 (0) 0
Erythema at TST site	2 (17) 2	0 (0) 0	0 (0) 0	3 (25) 4
Itching at TST site	1 (8) 1	0 (0) 0	0 (0) 0	3 (25) 4
Systemic AEs				
Any systemic AE	11 (92) 86	9 (75) 72	2 (67) 7	12 (100) 45
Headache	3 (25) 5	7 (58) 15	1 (33) 1	7 (58) 9
Sediment in urine^b^	5 (42) 5	3 (25) 5	1 (33) 2	5 (42) 6
Fever	2 (17) 2	3 (25) 4	1 (33) 1	3 (25) 3
Sweating	3 (25) 4	1 (8) 1	0 (0) 0	2 (17) 2
Anorexia	2 (17) 2	1 (8) 1	1 (33) 1	1 (8) 2
Weakness	1 (8) 1	2 (17) 2	0 (0) 0	2 (17) 2
Other (96 different categories)	10 (83) 67	9 (75) 44	1 (33) 2	7 (58) 21

^a^*n* signifies the number of participants experiencing the adverse event (AE) described

^b^Sediment refers to red and white blood cells, casts, bacteria, crystals and epithelial cells

*QFT* QuantiFERON-TB Gold-in-tube test, *TST* Tuberculin Skin Test

### Cell-mediated immunogenicity

**Fig. 2:**
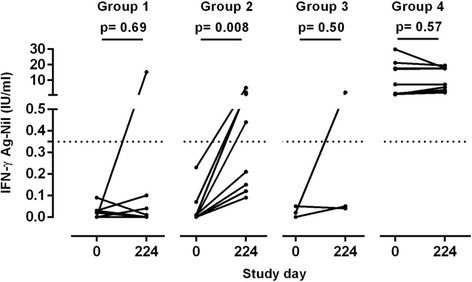
QuantifERON-TB Gold-in-tube assay conversion after H1/IC31® vaccinations. Blood samples for the Quantiferon-TB Gold-in-tube assay were taken prior to administration of the first H1/IC31® vaccination (study day 0) and at final visit (study day 224). Each dot indicates the IFN-γ concentration (IU/ml) for each study participant prior to, and after, study vaccinations in groups 1, 2, 3 and 4. A horizontal dotted line represents the cutoff value for Quantiferon positivity. The *p* values between day 0 and 224 were calculated by the Wilcoxon matched-pairs signed rank test

**Fig. 3:**
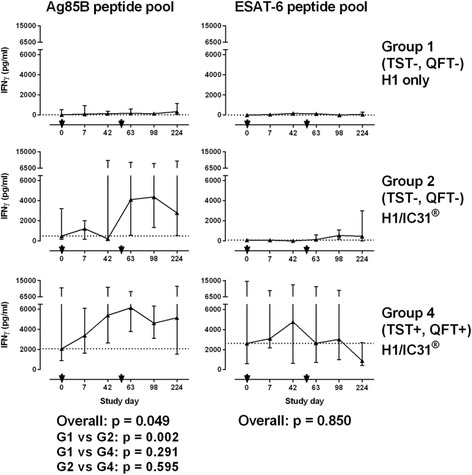
Longitudinal kinetics of H1-specific T cells measured by IFN-γ ELISA. Lines represent the median secreted IFN-γ levels in response to stimulation with Ag85B or ESAT-6 peptide pool for groups 1, 2 and 4. Error bars indicates interquartile range. Black arrows indicate vaccination time points. For each stimulation, area under the curve (AUC) values were compared using Kruskal-Wallis (overall effect) and if *p* < 0.05, Mann-Whitney tests were performed for comparison between individual groups

Blood samples for the QFT assay were drawn at baseline (day 0) and at the final visit day 224. As per inclusion criteria, all participants in groups 1, 2 and 3 were QFT negative at baseline and all participants in group 4 were QFT positive at baseline (Fig. 2). Among the Mtb-naïve participants in group 1, one participant out of nine (11%) converted QFT status after two H1 vaccinations without adjuvant. However, when the Mtb-naïve participants in group 2 were vaccinated with the adjuvanted and ESAT-6 containing H1/IC31® vaccine, a significant (*p* = 0.008) increase in IFN-γ responses was induced in all participants and four out of eight (50%) converted their QFT status. In group 3, one out of three participants (33%) converted. The Mtb-infected participants in group 4 had very high IFN-γ responses at baseline (as per inclusion criteria) and no change was observed. Figure 3 shows the longitudinal kinetics assessed by IFN-γ ELISA assay for the different stimulation conditions and groups. For Ag85B stimulation, vaccinations with H1/IC31® induced a significantly stronger response (as defined by a larger area under the curve (AUC)) compared to vaccinations without adjuvant IC31® (group 2 vs. group 1; *p* = 0.002). In contrast to the QFT assay, the IFN-γ ELISA assay only detected small immune responses after ESAT-6 stimulation. This may be due to either lower sensitivity of the assay or the fact that the QFT assay includes two additional antigens (CFP-10 and TB7.7). Baseline ESAT-6 and Ag85B responses were very pronounced in the already Mtb-infected participants in group 4, and little or no increase in the magnitude of responses were seen at the subsequent time points in this group. For this reason, no significant difference in ESAT-6 and Ag85B responses in AUC values were seen between group 4 and the other groups.

### Humoral immunogenicity

**Fig. 4:**
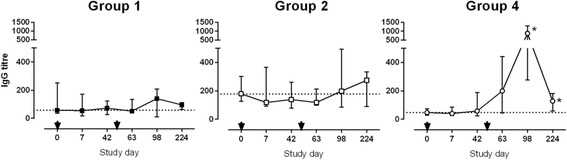
H1-specific IgG antibody titers. H1-specific IgG levels were measured before first vaccination (study day 0) and at five subsequent study days. Shown are the medians for groups 1, 2 and 4. Error bars represent the interquartile range. Black arrows indicate vaccination time points. A dotted line indicates IgG measured at baseline. For each group, vaccination time points were compared using Kruskal-Wallis (overall effect) and, if *p* < 0.05, Mann-Whitney tests compared each post-vaccination time point to baseline (day 0). Asterisks indicate a significantly increased titer in group 4 at study days 98 and 224 compared to baseline (*p* = 0.0002 and 0.024)

Plasma levels of IgG antibodies specific to H1 recombinant protein were measured at baseline and at five subsequent time points (Fig. 4). In the Mtb-naïve participants (groups 1 and 2), two vaccinations did not elicit a measurable IgG response. In the Mtb-infected group (group 4) a significant increase in IgG titer was seen in most participants 6 weeks after the second vaccination with H1/IC31® (study day 98; *p* = 0.0002) and at the final visit (study day 224; *p* = 0.024) compared to baseline titers.

## Discussion

We have completed a phase I, open-label clinical trial investigating the safety and immunogenicity of the H1/IC31® TB vaccine in 27 Mtb-uninfected and 12 Mtb-infected volunteers living in a highly TB-endemic area. The trial was planned to include a total of 48 participants, but after a prolonged period of screening, we decided to end enrollment after 39 individuals were recruited. There were two main reasons for the prolonged screening phase. First, no country-specific normal ranges for blood parameters existed and a set of international normal ranges for laboratory parameters were, therefore, used in the screening process. This resulted in a larger than expected number of individuals not being eligible due to eosinophil counts being higher than the expected range. The high eosinophil counts observed were not suspected to be associated with abnormal physiological conditions, but probably reflect a history of exposure to helminth infections or seasonal allergic responses, which are common in the general population at the trial site [18]. As active helminth infection may interact and exacerbate Mtb-related immune responses, it was important to exclude such volunteers from the trial [19]. This emphasizes the importance of establishing appropriate normal ranges for laboratory parameters in general and for eosinophil counts specifically, prior to conducting trials in non-western populations. Second, very few persons in the population were able to provide the necessary combination of a positive TST test (≥10 mm) and a negative QFT test for inclusion in trial group 3. This was probably due to underestimation of the waning of BCG-induced TST positivity in the absence of Mtb infection [20, 21]. After a prolonged screening phase, recruitment was stopped because of the limited remaining shelf-life of the vaccine even though only three volunteers had been found eligible to be included in group 3 at the time.

Two serious adverse events (SAEs) were reported in this study. Both volunteers were followed in accordance with the protocol and recovered without sequelae within 72 h. In both cases, the concern was raised laboratory blood parameters; aspartate aminotransferase (AST) and alanine aminotransferase (ALT) in the first case and creatine phosphokinase (CPK) elevation in the second. As both events were reported as possibly related to the vaccine, these events were important findings. As well, previous trials conducted in the Netherlands with H1 vaccine did not have any SAEs [14, 15].

Previous reports on the H1/IC31® vaccine have mainly reported local AEs, such as stiffness, erythema, induration, pain or tenderness at the injection site, and systemic events such as fever, headache, fatigue or myalgia [14, 15]. These mentioned AEs were also the most common in the present trial. We found more local AEs in Mtb-infected individuals compared to Mtb-naïve individuals. We also observed five participants who experienced erythema and/or itching not at the vaccine injection site, but at the site of the TST test used in the screening. In one case, the reaction was seen as cause enough to withdraw the volunteer from the study prematurely. The five participants with reactions at the previous TST site did not stand out from the remaining participants with regard to immunogenicity measures or other safety parameters. Overall, the H1/IC31® vaccine was well tolerated not only in Mtb-naïve, but, more importantly, in already Mtb-infected individuals; and apart from the two SAEs described above; most reported AEs were mild and in general resolved within a week. Overall evaluation of the safety of H1/IC31® has allowed the initiation and completion of two further clinical trials in TB-endemic areas with the vaccine. Although the participants in this trial were all male, previous trials have shown the safety of the vaccine in women participants as well [15].

T-cell responses to the vaccine were observed by the increase in IFN-γ responses to ESAT-6, TB7.7 and CFP-10 as measured by the QFT assay and to Ag85B and ESAT-6 in the IFN-γ ELISA assay. Whether the conversion in QFT assay observed is due to the vaccine or acquired infection cannot be differentiated; however, since the exposure to Mtb is the same in both study groups, it is likely that the stronger immune response and QFT conversion observed in participants vaccinated with the adjuvant is due to the vaccine. QFT conversion was also observed in the previous H1 trial; however, it was transient and either reverted or decreased in intensity at 2.5 years post vaccination [15]. In both assays, responses were low when the H1 fusion protein was injected alone, whereas the adjuvanted H1/IC31® vaccine induced a significantly higher immune response, confirming the need for adjuvant in a subunit vaccine, even in individuals living in a TB-endemic area. This is in agreement with a recent trial investigating the H1 antigen given with or without the adjuvant CAF-01, where a similar clear difference was observed [22]. In this trial, ESAT-6 responses were lower compared to Ag85B in Mtb-naïve vaccine recipients, whereas individuals with an already established Mtb infection had much more pronounced responses to ESAT-6. This is also in accordance with previous findings [14–16, 22, 23]. This highlights that using only measurement of IFN-γ to assess T-cell immunogenicity is insufficient in already Mtb-infected individuals. In such cases, more sophisticated immunogenicity assays are needed in order to differentiate between pre- and post-vaccination responses. Previously Mtb-infected individuals were also included in a previous H1/IC31® trial in the Netherlands, but showed much more limited baseline responses [15]. The reason for this difference is most likely due to repeated exposure to Mtb in the Ethiopian study participants in the present trial. It may also reflect the fact that TB patients in the Netherlands tend to be diagnosed and treated earlier in the course of their disease than Ethiopian patients, thus limiting the development of antigen-specific responses and highlights the importance of also conducting early phase vaccine trials in Mtb-endemic areas such as Ethiopia. Indeed, the general immune state of individuals living in highly TB-endemic areas is likely to be very different from that of areas where TB is not endemic. This possibility is supported by the high eosinophil counts found in many of the participants screened in this trial.

Previously reported trials with H1/IC31® saw no, or very limited, antibody responses in Mtb-naïve individuals but significant responses in Mtb-infected participants [14, 15, 22]. This was also seen in the trial reported here, where we observed IgG antibodies against the vaccine in the plasma after two vaccinations, but only in the group of already Mtb-infected participants. Most recently, similar results were seen with the H56/IC31®vaccine where two vaccinations induced an anti-H56 IgG response in 60% of Mtb-infected individuals but only in 10% of Mtb-naïve participants [23]. Of interest is that in the latter trial, a third vaccination raised the response to 60% in the Mtb-naïve participants as well.

A limitation of this study is the low sample size, but, even so, the successful inclusion of the Mtb-infected participants from a highly TB-endemic area and the acceptable safety profile of the vaccine in that population has enabled the vaccine to progress to a larger phase II trial investigating dose, safety and immunogenicity of the H1/IC31® vaccine in a target population of 240 adolescents from South Africa, of which half were Mtb-infected at inclusion.

## Conclusions

In summary, we investigated the TB vaccine candidate H1/IC31® in both Mtb-naïve and Mtb-infected individual living in a highly TB-endemic area. The vaccine was safe and generally well tolerated. Two SAEs were reported in Mtb-infected participants where a relationship to the vaccine could not be excluded. The trial also confirmed the need for an adjuvant and highlighted the importance of early phase testing of novel TB vaccine candidates in TB-endemic areas.

## Additional file

Additional file 1: Contains data of Physical exam, Hematology, Biochemistry, QuantiFERON TB Gold assay, IFNγ ELISA, and IgG ELISA results. (XLSX 46 kb)

## Abbreviations

Ag85: Antigen 85
AE: adverse event
AHRI: Armauer Hansen Research Institute
ALERT: All Africa Leprosy Rehabilitation and Training Centre
ALT: Alanine aminotransferase
AST: Aspartate Aminotransferase
AUC: Area under the curve
BCG: Bacillus Calmette-Guérin
CFP-10: Culture Filtrate Protein-10
CPK: Creatinine phosphokinase
CRF: Case Record Form
DACA: Drug Administration and Control Authority
DSMB: Data Safety Monitoring Board
EDCTP: European and Developing Countries Clinical Trials Partnership
ELISA: Enzyme Linked Immunosorbent Assay
ELISPOT: Enzyme Linked Immunospot Assay
ESAT-6: 6-kDa early secretory antigenic target
FMHACA: Food, Medicine and Health Care Administration and Control Authority of Ethiopia
GSK: Glaxo-Smith Kline
HBV: Hepatitis B virus
HCV: Hepatitis C virus
HIV: Human immunodeficiency virus
ICL: International Clinical Laboratories
IU: International unit
Mtb: *M. tuberculosis*
PBMC: Peripheral blood mononuclear cells
QFT: QuantiFERON-TB Gold in-tube test
SAE: Serious adverse events
SSI: Statens Serum Institut
TB: Tuberculosis
TST: Tuberculin Skin Test
